# Rare Combination of Partial ARCAPA and Dual LAD: Insights into Complex Coronary Variants

**DOI:** 10.3390/diagnostics16060886

**Published:** 2026-03-17

**Authors:** Chiara Morelli, Francesca Troise, Alessia Spitaleri, Sterpeta Guerra, Nicola Maggialetti

**Affiliations:** Interdisciplinary Department of Medicine, Section of Radiology and Radiation Oncology, University of Bari “Aldo Moro”, 70124 Bari, Italy

**Keywords:** computed coronary tomography angiography (CCTA), rare congenital coronary artery anomaly, dual left anterior descending artery (dual LAD), anomalous right coronary artery from the pulmonary artery (ARCAPA)

## Abstract

This case report highlights the coexistence of two rare coronary artery anomalies assessed by computed coronary tomography angiography (CCTA). We present the case of a 51-year-old hypertensive patient with a type II double left anterior descending artery (LAD) and an anomalous infundibular branch originating from the pulmonary artery (partial ARCAPA). This association may have contributed to a limited ischemic burden, explaining the patient’s asymptomatic state. Knowledge of these rare coronary anatomies is essential for accurate diagnosis and management.

**Figure 1:**
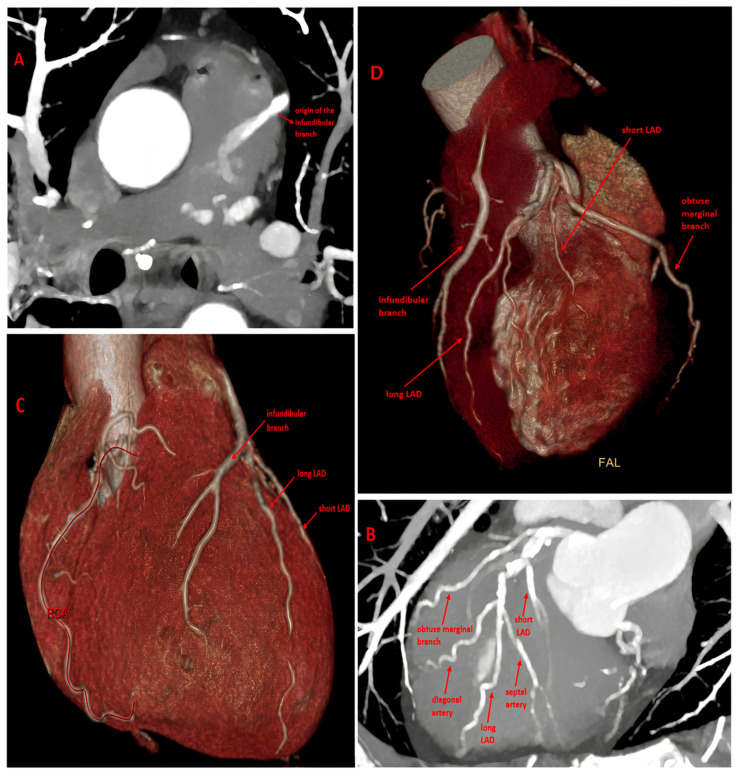
CT evaluation of the coronary artery anatomy. (**A**) MIP Coronary Computed Tomography Angiography (CCTA) image in the axial plane shows the abnormal origin of the dilated infundibular artery from the main pulmonary artery (partial ARCAPA), with evidence of contrast enhancement reflow. (**B**) MIP CCTA image in the oblique plane shows the dual LAD variant originating from the LAD proper. (**C**,**D**) VR images show the infundibular coronary perfusion territory and the anatomical configurations of the two anomalies. ARCAPA is extremely rare, with an estimated incidence of approximately 0.002% in the general population and accounting for only about 0.12% of all coronary artery anomalies [1]. Dual LAD is relatively more common, with a prevalence ranging from approximately 1% in conventional angiographic series to about 4–6% in large CCTA cohorts [2]. However, the coexistence of these two anomalies is considered exceptionally rare. In fact, the literature is limited to isolated case reports, and to the best of our knowledge, no large epidemiological studies report the prevalence of their simultaneous occurrence [3]. Our focus is on a hypertensive 51-year-old male patient with hypercholesterolemia; the electrocardiogram showed sinus rhythm at 75 bpm with diffuse repolarization abnormalities, including T-wave inversions in the precordial leads, consistent with a left ventricular strain pattern in the context of systemic hypertension. Transthoracic echocardiography showed a left ventricle of normal size with mildly increased wall thickness and preserved systolic function (LVEF 55%). The left atrium was mildly dilated, while the right sections were within normal limits. The aortic valve was tricuspid with thickened cusps but no stenosis. The mitral and tricuspid valves were mildly thickened with trivial to mild regurgitation and no stenosis. No pericardial effusion was observed. Doppler assessment demonstrated an E/A ratio < 1, consistent with mild diastolic dysfunction. Given the patient’s asymptomatic status, cardiovascular risk factors, and nonspecific findings on ECG and echocardiography, CCTA was performed as the initial noninvasive test to assess both coronary artery disease and congenital coronary anatomy [2,4]. The findings included a split LAD deriving from the LAD proper (type II in Spindola-Franco’s Classification [2,5,6] and an infundibular artery, usually a right branch, that anomalously originates from the left side of the main pulmonary artery [Figure 1]. Typically [1,3], the artery originating from MPA is dilated and tortuous due to collateral burden and retrograde diastolic flow toward the MPA, evidenced by a blush of contrast enhancement. Furthermore, this artery does not follow the typical course of the right coronary artery and supplies the anterior walls of both the left and right ventricles. The ostium of the right coronary artery originates from the aorta; however, the RCA follows the acute margin and does not reach the posterior interventricular sulcus or several segments of the right ventricular wall. Coronary circulation is, in fact, left-dominant, with the PDA and PL branches supplied by the circumflex artery. No myocardial scarring was evident on CCTA. The coexistence of a dual LAD and the limited myocardial territory supplied by the ARCAPA may reduce the overall ischemic burden and could explain the patient’s asymptomatic status, as well as the normal ECG and echocardiography findings [7,8,9]. Consequently, no dedicated functional ischemia testing was performed. In this asymptomatic patient, a conservative approach with close clinical follow-up was chosen, including noninvasive assessments such as ECG, Holter monitoring, and transthoracic echocardiography every six months. Long-term surveillance is therefore recommended to monitor for the development of potential subclinical myocardial dysfunction and to better understand the natural history of the anomaly. This case highlights the uniqueness of this association, the importance of individualized, anatomy-based management, and the value of detailed imaging assessment [10].

## Data Availability

The original data presented in this study are included in the article. Further inquiries can be directed to the corresponding author.

## References

[1] Balakrishna P., Illovsky M., Al-Saghir Y.M., Minhas A.M. (2017). Anomalous Origin of Right Coronary Artery Originating from the Pulmonary Trunk (ARCAPA): An Incidental Finding in a Patient Presenting with Chest Pain. Cureus.

[2] Maggialetti N., Greco S., Lorusso G., Mileti C., Sfregola G., Brunese M.C., Zappia M., Belfiore M.P., Sullo P., Reginelli A. (2023). The Role of Coronary CT Angiography in the Evaluation of Dual Left Anterior Descending Artery Prevalence and Subtypes: A Retrospective Multicenter Study. J. Pers. Med..

[3] Nakamae K., Ichihara Y., Morita K., Niinami H. (2021). Surgical management for dual left anterior descending artery with anomalous origin of left coronary artery from pulmonary artery: A case report. Gen. Thorac. Cardiovasc. Surg..

[4] Ajam A., Rahnamoun Z., Sahebjam M., Sattartabar B., Razminia Y., Tafti S.H.A., Hosseini K. (2022). Cardiac imaging findings in anomalous origin of the coronary arteries from the pulmonary artery; narrative review of the literature. Echo Res. Pract..

[5] Jariwala P., Jadhav K.P., Koduganti S. (2021). Dual left anterior descending artery: Diagnostic criteria and novel classification. Indian. J. Thorac. Cardiovasc. Surg..

[6] Kalekar T., Pachva A., Kumar S.P. (2024). Duplication of Left Anterior Descending Artery: A Case Report on a Rare Abnormality. Cureus.

[7] Gutti R., Pilla A., Vasireddy S. (2025). Anomalous origin of the right coronary artery from the pulmonary artery (ARCAPA) unmasked by coronary artery disease of the left coronary system in a 62-year-old man. Indian. J. Thorac. Cardiovasc. Surg..

[8] VanLoozen D., Bykhovsky M.R., Kapoor D., Bates W.B., Murdison K.A., Polimenakos A.C. (2019). Myocardial ischemia and anomalous origin of the right coronary artery from the pulmonary artery in the adult: Management implications and follow-up. World J. Pediatr. Congenit. Heart Surg..

[9] Nishino S., Watanabe N., Komatsu M., Yano M., Shibata Y. (2020). Anatomical and physiological assessment of a symptomatic anomalous origin of the right coronary artery from the pulmonary artery by noninvasive imaging examinations. J. Cardiol. Cases.

[10] Kappel C., Chow J., Ahmed Z., Schwalm J.D., Amin F. (2021). Cardiogenic shock in the context of newly diagnosed anomalous origin of the right coronary artery originating from the pulmonary artery: A case report. Eur. Heart J. Case Rep..

